# Salivary cortisol in healthy dogs: a randomized cross-over study to evaluate different saliva stimulation methods and their effects on saliva volume and cortisol concentration

**DOI:** 10.1186/s12917-021-02890-1

**Published:** 2021-05-17

**Authors:** Solène Meunier, Michael Groessl, Claudia Reusch, Felicitas Boretti, Nadja Sieber-Ruckstuhl

**Affiliations:** 1 Clinic for Small Animal Internal Medicine, Vetsuisse Faculty, University of Zurich, Zurich, Switzerland; 2 Department of Nephrology and Hypertension, Inselspital, Bern University Hospital, University of Bern, Bern, Switzerland

**Keywords:** Ginger, Cotton swab, Saliva, Canine, Cushing’s syndrome, Cortisol measurement, LC-MS

## Abstract

**Background:**

Salivary cortisol collected at home is a useful test to diagnose and monitor Cushing’s syndrome in humans. The main problem in dogs is to retrieve a sufficient amount of saliva. The aim of this study was to evaluate different salivary collection methods and compare their effects on volume, pH and cortisol concentration of saliva. Sixteen healthy Beagles were used in a 4 × 4 randomized crossover study with a washout period of 1 week between each of the following collection methods: 1. Salimetrics® cotton swab dipped in ginger powder (ginger group); 2. beef-flavored Salimetrics® (bouillon group); 3. Salivette® cotton swab with an enclosed treat (treat group); 4. plain Salimetrics® (control group). First, baseline saliva (plain cotton swab, S0) and, 2 min later, experimental saliva (according to group allocation above, SExp) were collected. Saliva was gathered by holding the swabs in the animal’s mouth for 2 min. After the cross-over study, another saliva sample was collected from all dogs by the ginger method, using a 30 s sampling time (30s-ginger method). Cortisol concentrations were measured by liquid chromatography tandem mass spectrometry.

**Results:**

All three stimulation methods increased saliva production significantly (S0 compared to SExp: ginger *p* = 0.0005; bouillon *p* = 0.009; treat *p* = 0.007). Only ginger stimulation, however, generated a significantly higher amount of saliva (SExp) compared to the control group (*p* = 0.00001; median (range) amount of saliva for SExp: ginger 1200 ul (600–1700), bouillon 650 ul (200–1900), treat 700 ul (300–1000), control 400 ul (0–1100)). The amount of saliva retrieved by the 30s-ginger method was still higher than that from the control group (*p* = 0.0004). Bouillon and treat stimulation led to decreased pH values (bouillon, *p* = 0.0028; treat, 0.0018). Excitement was higher in the ginger group (*p* = 0.01). Chewing was intensified in the ginger and treat group (ginger, *p* = 0.003; treat, 0.0009). The cortisol concentration SExp was higher compared to that of S0 in the ginger and treat group (*p* = 0.02, 0.003). The experimental cortisol concentrations (SExp) were not different between groups.

**Conclusions:**

The 30s-ginger method could prove useful in evaluating or monitoring dogs with Cushing’s syndrome, as sampling at home for 30 s by the owner seems feasible.

## Background

Salivary cortisol is in equilibrium with the biologically active free cortisol in the blood and does not seem to be affected by the rate of saliva production [1]. A rise in blood cortisol, results in an increase in salivary cortisol within minutes [2]. Determination of late-night salivary cortisol (LNSC) is an established screening test for the diagnosis of Cushing’s syndrome in human patients [1, 3]. Saliva is collected on two separate evenings at home either by passive drooling or by chewing a swab for about 60–120 s [1, 3, 4]. LNSC is furthermore an excellent method for monitoring Cushing’s disease patients post-operatively for surgical failure or recurrence, and was found to perform better than urinary free cortisol or early postsurgical morning plasma cortisol [5, 6]. Saliva collection is a straightforward, minimally invasive procedure in adults; however, in infants several studies reported difficulties in obtaining a sufficient amount of material [7, 8].

In dogs, salivary cortisol has been mainly used as a measure of stress response, because saliva collection is less invasive than blood sampling [9]. However, as with infants, it can be difficult to collect a sufficient amount of saliva in dogs, due to their unwillingness to chew the collection material, which was the major problem recorded in the first clinical study on using salivary cortisol to diagnose Cushing’s syndrome in dogs [10]. In 27% of the samples of healthy dogs and 50% of the samples of dogs with Cushing’s syndrome an insufficient amount of saliva was obtained [10]. To overcome this problem, several methods for stimulating saliva production in dogs have been described [9, 11–15]. Citric acid has been most widely used, either by sprinkling a few pellets or crystals onto the dog’s tongue or by swabbing the dog’s mouth and gums with a citric acid-soaked cotton ball [11–15]. Other substances or methods used were acetic acid, sucrose, sodium chloride, beef-flavored cotton ropes or beef-flavored hydrocellulose swabs [11, 12, 16]. One study in humans, reported the use of ginger with its pungent taste as a salivary stimulant [17]. So far, to the best of the authors’ knowledge, there have been no studies on using ginger as a salivary stimulant in dogs. A major drawback of some stimulants and some collecting materials is their known influence on hormone concentrations [12, 18–21].

All studies cited above used immunoassays to measure salivary cortisol in dogs. However, antibody-based immunoassays suffer from matrix effects through the interaction of sample components with the antibody-antigen binding property and show cross-reactivity with cortisol metabolites [22, 23]. In contrast, assays that directly measure cortisol such as liquid chromatography tandem mass spectrometry (LC-MS/MS) circumvent these problems. To our knowledge, the use of LC-MS/MS has not been reported in canine salivary cortisol studies.

Before saliva cortisol can be further assessed and finally used to diagnose or monitor Cushing’s syndrome in dogs, a more reliable collecting method has to be established. Therefore, the objective of this study was to evaluate three different salivary stimulation methods, including a ginger-based method, and to compare their effects on volume, pH and cortisol concentration of the saliva and on the behavior of the dogs during sampling. Salivary cortisol was determined by means of LC-MS/MS, making this the first report of the LC-MS/MS measuring method in dogs.

## Results

### Experimental study

#### Amount of saliva

**Fig. 1:**
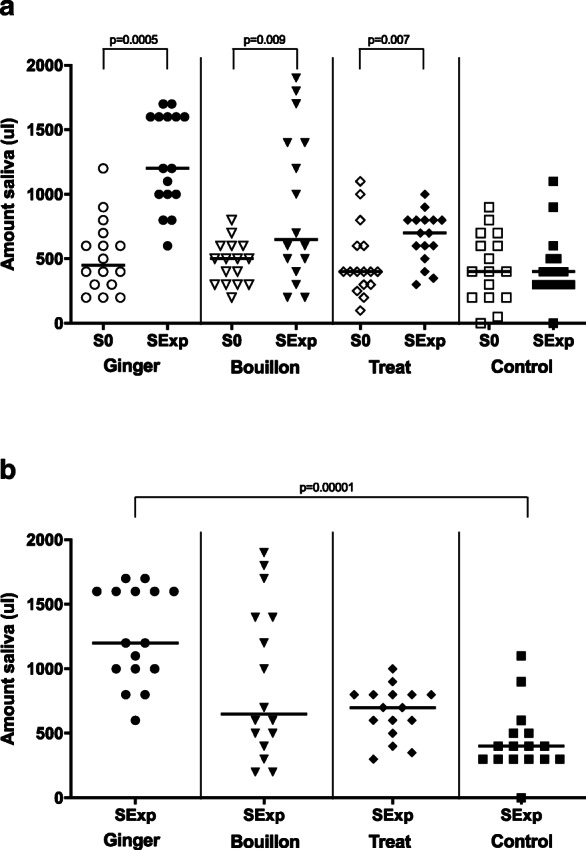
**a** Amount of baseline (S0) and experimental (SExp) saliva of the four groups. **b** Amount of experimental saliva (SExp) of the four groups. The horizontal line represents the median of each group. Clamps represent significant differences

The amount of experimental saliva (SExp) was significantly higher than the amount of baseline saliva (S0) in the ginger, bouillon and treat groups (Table 1, Fig. 1a; *p* = 0.0005, 0.009, 0.007, respectively). There was no increase in SExp compared to S0 in the control group (Table 1, Fig. 1a; *p* = 0.8). Only in the ginger group was the amount of SExp significantly higher than SExp of the control group (Table 1, Fig. 1b; *p* = 0.00001). There was one animal in the control group from which no saliva could be collected during the baseline and the experimental sampling.

**Table 1:** Data (median and range) of baseline saliva (S0) and experimental saliva samples (SExp) of the four groups

		Ginger			Bouillon		Treat		Control	
	Unit	S0 – 2 min	SExp – 2 min	SExp – 30s	S0	SExp	S0	SExp	S0	SExp
**Amount of saliva**	**ul**	450 (200–1200)	1200 ^a b^ (600–1700)	900 ^b c^ (400–1600)	500 (200–800)	650 ^a^ (200–1900)	400 (100–1100)	700 ^a^ (300–1000)	400 (0–900)	400 (0–1100)
**pH**	**Score** **1–5**	8 (8–9)	9 (8–9)		8.5 (7–9)	7 ^a b c^ (7–9)	9 (8–9)	7.5 ^a b c^ (6–9)	9 (8–9)	9 (8–9)
**Handling**	**Score** **1–5**	1 (1–2)	1 (1–2)		1 (1–3)	1 (1–3)	1 (1–5)	1 (1–5)	1 (1–2)	1 (1–2)
**Excitement**	**Score** **1–5**	1 (1–1)	1 ^a^ (1–2)		1 (1–3)	1 (1–3)	1 (1–4)	1 (1–5)	1 (1–1)	1 (1–1)
**Chewing**	**Score** **1–5**	2 (1–3)	3.5 ^a^ (1–4)		2 (1–4)	3 (1–5)	2 (1–4)	3 ^a^ (3–4)	2 (1–5)	2 (1–5)
**Saliva cortisol concentration**	**nmol/l**	0.4 (0.1–1.2)	0.7 ^a^ (0.5–3.6)	0.3 ^c^ (0.1–1.0)	0.4 (0.2–1.5)	0.4 (0–1-1.1)	0.4 (0.2–2.2)	0.6 ^a^ (0.3–2.4)	0.4 (0.1–1.2)	0.5 (0.2–1.7)

^a^Significant difference from S0

^b^Significant difference from SExp of the control group

^c^Significant difference from SExp Ginger 2 min

**Fig. 2:**
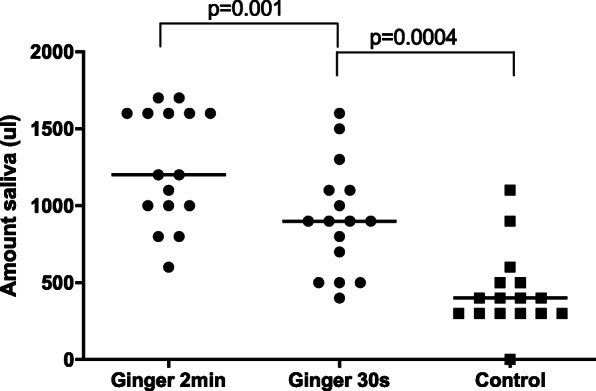
Amount of experimental saliva of the ginger, the 30s-ginger and the control group. The horizontal line represents the median of each group. Clamps represent significant differences

Comparing the two-minute ginger method with the 30s-ginger method revealed the collection of a significantly lower amount of saliva after 30s (Table 1, Fig. 2, *p* = 0.001). The lowest amount of saliva collected after 30s was 400 ul (1 dog). The amount of saliva collected with the 30s-ginger method was, however, still significantly higher than the amount collected in the control group (Table 1, Fig. 2, *p* = 0.0004).

#### pH

**Fig. 3:**
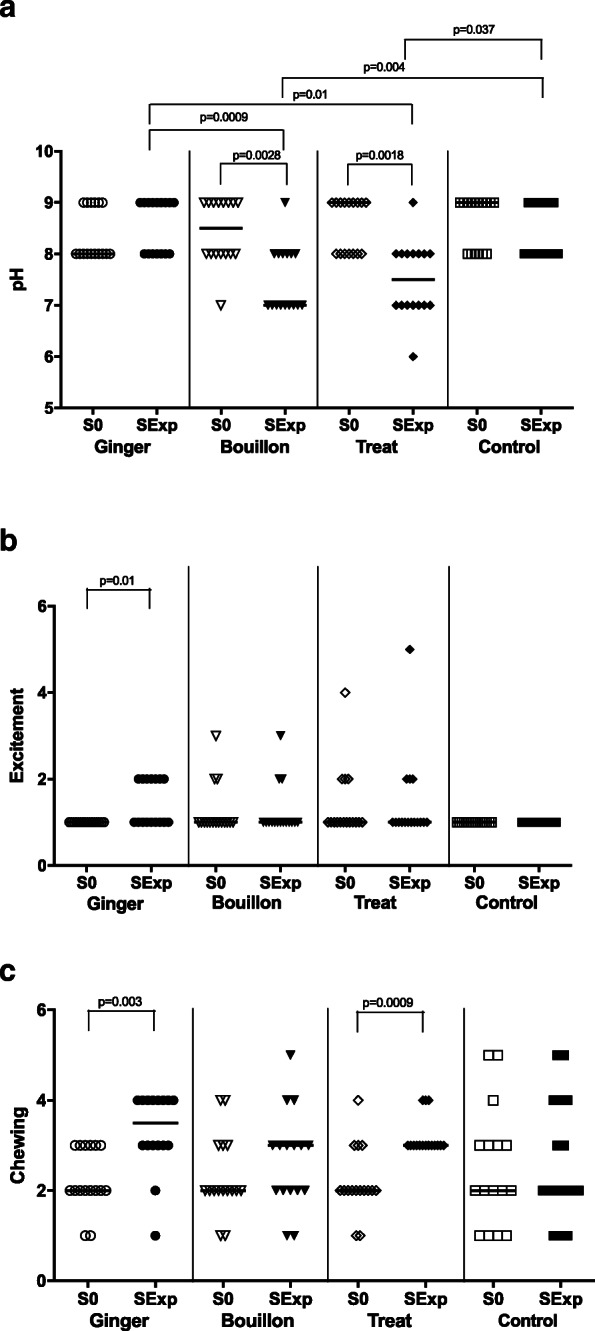
Scoring of pH (**a**), excitement (**b**) and chewing (**c**) of baseline (S0) and experimental (SExp) saliva of the four groups. The horizontal line represents the median of each group. Clamps represent significant differences

In the bouillon and the treat group the pH of SExp was lower than that of S0 (Table 1, Fig. 3a, *p* = 0.0028, 0.0018, respectively). The pH of SExp of the bouillon and treat group was also lower than that of the SExp of the ginger or control group (Table 1, Fig. 3a, *p* = 0.0009, 0.01, 0.004, 0.037, respectively).

#### Handling, excitement and chewing

There was no significant difference in the handling scores in any group (Table 1). In the ginger group, the score for excitement was significantly higher during collection of SExp than during collection of S0 (Table 1, Fig. 3b, *p* = 0.01). In the ginger and treat groups, the score for chewing was significantly higher during collection of SExp than during collection of the S0 (Table 1, Fig. 3c, *p* = 0.003, 0.0009, respectively).

#### Validation results for measurement of salivary cortisol

The results of inter-day and intra-day accuracy and precision measurements are summarized in Table 2. The calibration curves demonstrated linearity (r^2^ > 0.99) and the lower and upper limit of quantification were defined as 0.01 and 300 ng/mL respectively, (0.028 and 827 nmol/L, respectively). Salivary canine cortisol concentrations determined by internal calibration compared to standard addition experiments showed an average error of 6.7% (*n* = 4), thus proving the precision of the method. Salivary cortisol levels of canine samples showed < 10% RSD (triplicates of 4 independent samples) and therefore good reproducibility.

**Table 2:** Accuracy and precision of the method assessed at four different levels

	0.3 ng/ml	3 ng/ml	7 ng/ml	100 ng/ml
**Inter-day accuracy (RSD, %)**	5.3	6.9	0.9	1.2
**Inter-day precision (RSE, %)**	1.2	−4.3	2.2	2.5
**Intra-day accuracy (RSD, %)**	5.8	6.9	7.8	6.1
**Intra-day precision (RSE, %)**	4.3	−7.2	0.9	3.4

#### Cortisol concentrations in saliva

**Fig. 4:**
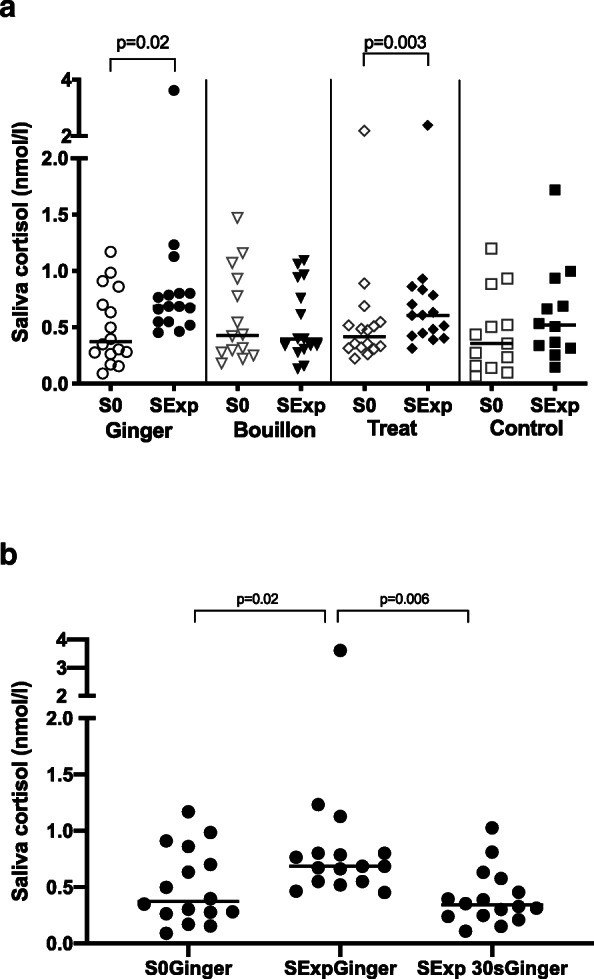
**a** Cortisol concentrations of baseline (S0) and experimental (SExp) saliva of the four groups. **b** Cortisol concentrations of baseline ginger (S0Ginger), experimental ginger (SExpGinger) and experimental 30s-ginger (SExp30sGinger) saliva. The horizontal line represents the median of each group. Clamps represent significant differences

The cortisol concentration of SExp was significantly higher compared to that of S0 in the ginger and treat groups (Table 1, Fig. 4a; *p* = 0.02, 0.003, respectively). The cortisol concentration of SExp 30s-ginger was significantly lower than that of SExp ginger (Fig. 4b, *p* = 0.006), but not significantly different from S0 ginger (Table 1, Fig. 4b). There was no significant difference in the cortisol concentrations in S0 or SExp between the four groups (Table 1, Fig. 4a).

#### Test for acclimatization

**Fig. 5:**
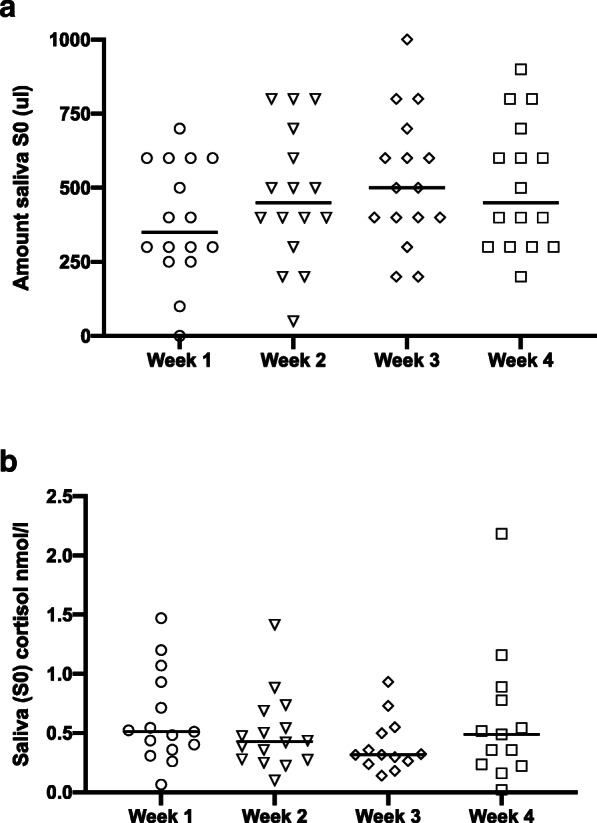
**a** Amount of baseline saliva (S0) per week of collection. The horizontal line represents the median of each group. **b** Cortisol concentration of the baseline saliva sample (S0) per week of collection. The horizontal line represents the median of each group

There was no significant difference between the amounts of saliva collected or the cortisol concentrations across the 4 weeks (Fig. 5a and b).

#### Cortisol concentrations in serum and comparison to saliva cortisol concentrations

**Fig. 6:**
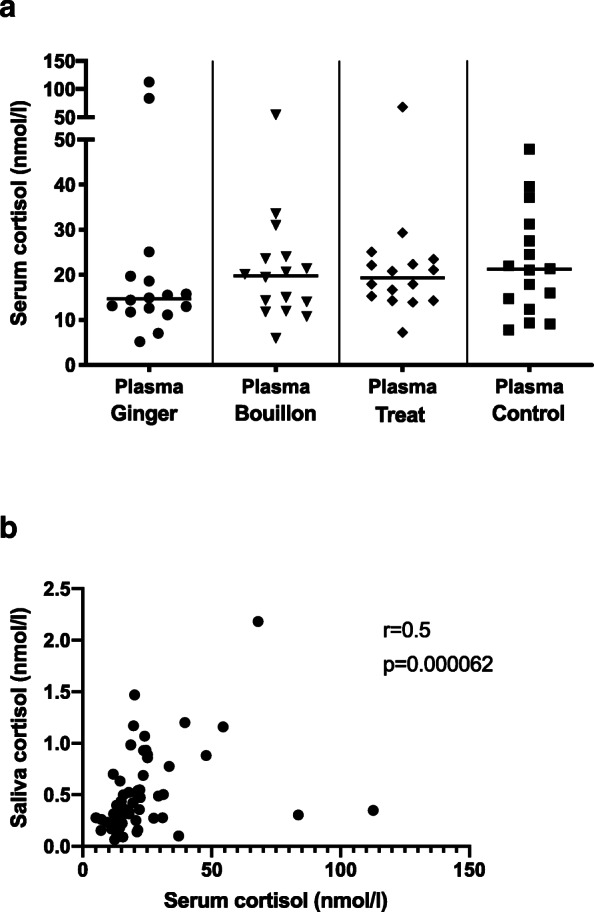
**a** Serum cortisol concentrations of the four groups. The horizontal line represents the median of each group. **b** Correlation between serum and baseline salivary cortisol concentration

The cortisol concentration in serum did not differ significantly across the four groups (Table 3, Fig. 6a). The baseline salivary cortisol concentration (S0) correlated significantly with the serum cortisol concentration (Fig. 6b, *p* = 0.000062, r = 0.5). The salivary cortisol concentration represented between 0.3 and 7.3% (median: 2.2%) of that of the serum cortisol concentration.

**Table 3:** Saliva and serum cortisol concentrations (median and range) of the four groups

		Ginger	Bouillon	Treat	Control
	Unit				
**Saliva cortisol concentration – S0**	**nmol/l**	0.4 (0.1–1.2)	0.4 (0.2–1.5)	0.4 (0.2–2.2)	0.4 (0.1–1.2)
**Serum cortisol concentration**	**nmol/l**	15 (5–113)	20 (6–54)	20 (7–68)	21 (8–48)

## Discussion

In the present study, we were able to show that significantly more saliva could be gained using an enriched cotton swab with either ginger, bouillon or treat, compared to using a plain cotton swab in the same dog. However only the ginger-dipped method led to an increased amount of saliva in the experimental sample compared to the experimental sample of the control group. For the determination of cortisol in saliva a sufficient amount of saliva is mandatory. This was the major limitation in an earlier study measuring salivary cortisol in dogs. Salivary flow was severely reduced in dogs with Cushing’s syndrome in particular, so that the required amount of saliva could be obtained in only 50% of the dogs [10]. It is therefore imperative to use a reliable saliva-collection method in an experimental setting testing dogs for suspected Cushing’s syndrome. In our present study, all three of the stimulation protocols led to increased salivary flow, and thus the necessary minimal amount of saliva to measure cortisol concentration could be obtained.

The sampling act itself can be challenging in dogs, as unwillingness to chew can result in defensive movements, growling or even biting. Even trained veterinarians and technicians experienced difficulties keeping the collecting swab in a dog’s mouth for as long as 2 min to ensure collection of an adequate amount of saliva [10]. We therefore tried to reduce the collecting time: The method that provided the greatest amount of saliva was the ginger-dipped method; this method was therefore modified to a reduced collecting time of only 30 s, a time span during which it seemed reasonable to require owners to keep their dogs calm with a swab in their mouths. In the experimental dogs, this 30-s ginger-dipped method provided a sufficient amount of saliva in all dogs (> 400 ul). Hence, we believe that the short ginger method could be suitable for dog owners collecting saliva at home.

The sampling of saliva with a beef-flavored Salimetrics® or treat-enriched Salivette® led to a significant decrease in pH from 8.5 to 7 or 9 to 8, respectively. A drop in pH was shown to cause a significant increase in measured salivary cortisol concentration [12, 21]. This phenomenon, however, could not be observed in our study. The most likely explanation is that the decrease in pH in the bouillon and treat group was too small to influence cortisol concentrations. In the studies above, only a decrease in pH to < 4 caused a significant increase in cortisol concentrations [12, 21]. Another explanation could be the different assays used for measuring cortisol. In our study we used LC-MS/MS to determine cortisol concentrations, in contrast to the commonly used immunoassays. Steroid measurements by LC-MS/MS does not rely on antigen binding and is less influenced by sample properties [24, 25].

The ginger-dipped swab seemed to cause slightly more excitement in the dogs than the other two methods. This was most likely due to the pungent taste of ginger powder. Ginger contains many bioactive components. The primary pungent ingredient is 6-gingerol, which is believed to have a variety of remarkable pharmacological and physiological effects e.g. alleviation of nausea and vomiting [26]. Pungent 6-gingerol was shown to increase saliva flow in healthy humans by up to 60% [17]. The excitement observed in dogs chewing the ginger-dipped swab was short-lived; they seemed to react strongly to the first pungent taste and calmed down thereafter. Therefore, overall handling of the animals during the 2-min collecting time was not significantly different across the four groups. Chewing showed significant increase in the ginger and treat groups. Again, the pungent taste of 6-gingerol and the increased saliva flow seemed responsible for this in the ginger group. In the treat group, the animals were able to smell the treat enwrapped in the swab, which most likely led to intensified chewing movements. In contrast to the ginger group, however, the intensified chewing of the treat group did not result in an increased amount of saliva. One reason for this discrepancy could be the different collecting material used. In the treat group saliva was collected with a Salivette® held with a pair of clamps [10]. With this method it was clearly more difficult to keep the swab in the dog’s mouth for 2 min. Furthermore, hurting the animal with the metallic clamp was a possible risk. Using a different collecting material in the treat group had a practical reason: the Salimetrics® swabs used in the ginger, bouillon and control group were too small and could not be cut in half to enclose a treat.

The cortisol concentrations of the experimental samples were significantly higher than in the baseline samples from dogs in the ginger and treat group. The concentration of free cortisol in saliva reacts quickly to changes in serum cortisol and establishes an equilibrium within 5 min [27, 28]. One explanation for the increased cortisol concentrations of the experimental samples in the ginger and treat group could therefore be increased excitement during sampling. This was observed with the ginger-dipped swap, where the pungent taste seemed to briefly increase the dogs’ excitement. As the sampling period was 2 minutes this seemed enough time to affect the saliva cortisol concentration. A shorter sampling time would therefore be beneficial, hopefully reducing this influence and the increase in cortisol. This was confirmed by comparing the cortisol concentrations of the 30-s ginger sampling method with the baseline sample from the 2-min ginger sampling method. A shorter sampling period with a ginger-dipped swab did not lead to significantly increased cortisol concentrations. During treat sampling the dogs seemed also mildly excited. It seems possible, that the prospect of receiving a treat might have led to excitement and increased cortisol concentrations.

The increase in cortisol concentration in the experimental ginger and treat samples did not appear to be very high, because comparing the cortisol values of the experimental samples from all four groups revealed no difference. It is likely that the difference was diminished as the experimental sample of the control group was also slightly higher than the corresponding baseline value. Since the dogs remained in the examination room between collection of the baseline and experimental samples, it seems possible that the two-minute waiting period between sample-taking led to increased excitement and slightly increased cortisol concentrations. This two-minute waiting period was chosen to ensure that the first saliva sampling (baseline sample) did not influence the second sampling (experimental sample). In future studies, however, this waiting period should be shortened to circumvent increased excitement and possibly increased cortisol concentrations.

To evaluate whether dogs become used to saliva sampling, we analyzed amount of saliva collected and cortisol concentration per week. If the dogs had been more excited in the first week of sampling and less so in the last week, a significant difference in the amount of saliva or the cortisol concentration would be expected. However, we could not detect any differences between weeks and, thus, no evidence of adaptation to the sampling procedure.

The correlation between serum cortisol and saliva cortisol (S0) was significant but surprisingly low (r = 0.5). However, various studies in dogs have likewise shown a wide range of correlation coefficients (0.44–0.83) [27, 29].

LC-MS/MS was shown to be a robust method for the determination of saliva cortisol in dogs. The method shows linear response and is very accurate also at low concentrations. In future studies, the inclusion of additional steroids in the method which can be measured by LC-MS/MS in parallel should be evaluated.

This study has several limitations. First, in one group (treat) a different collecting swab (Salivette®) and collection technique was used (pair of clamps). The collecting swab (Salimetrics®) used in the ginger, bouillon and control groups was too thin to incorporate a treat. Second, it would have been ideal to evaluate the shorter collecting period (30s) with all three different saliva stimulating methods. This should be considered in a future study. In addition, the two-minute waiting period between the collection of the baseline sample (S0) and the collection of the experimental sample (SExp) seems long. Most likely, a shorter waiting period (e.g. 30s) would have been enough to exclude interference between the two sampling procedures. Further, the mild excitement observed in the treat group was not detected with the scoring system. Possibly, the scoring system had too few points of scores to detect mild excitement. Furthermore, jugular venipuncture at S0 may have produced a stressful situation. However, the dogs included were research beagles which are used to jugular venipuncture. Therefore the author believe that blood drawing did not lead to much stress in these dogs. Finally, an important aspect which should be considered in future studies is the collecting material for saliva: it could be shown in dogs that cotton wool swabs are very absorbent and did not allow the saliva to be released [30]. Replacing the swab by a sponge could resolve this problem, which could be shown in recent studies evaluating other biomarkers in canine saliva [31, 32].

## Conclusions

To conclude, this study shows that a ginger-dipped swab can stimulate saliva flow so intensively that a collection time of 30s is sufficient to retrieve the amount of saliva required for cortisol measurements. Despite brief excitation among the dogs in the ginger group, cortisol concentrations were not significantly different from those of the other groups. On the basis of our results we believe that the 30s-ginger method could be a valuable tool for evaluating or monitoring clinical dogs with Cushing’s syndrome; with this method, owners could sample their dog’s saliva at home in the accustomed environment, thus avoiding hospital stress.

## Material and methods

### Animals and ethics statement

The study design and protocols were reviewed and approved by the Cantonal Veterinary Office of Zurich (permission number ZH168/16). All applicable national, and/or institutional guidelines for the care and use of animals were followed.

The experimental study was performed using 16 purpose-bred beagles. The dogs were housed at a university facility in standard kennels in groups of 4, fed dry adult maintenance dog food, and given access to water ad libitum. There were 9 intact males and 7 intact females between 3 and 10 years of age (median, 6) and weighing between 10.4 and 19.9 kg (median, 14.3). All dogs were considered healthy on the basis of results of physical examination, cell blood count, serum biochemical analysis. All procedures were performed in the living environment and by the same investigator to control for possible situational biases on cortisol measurement, as recommended in previous studies [33]. Dogs were fasted overnight before blood and salivary samples were obtained to avoid possible effect of food contamination on the measurement.

### Study design

#### Experimental part of the study

**Fig. 7:**
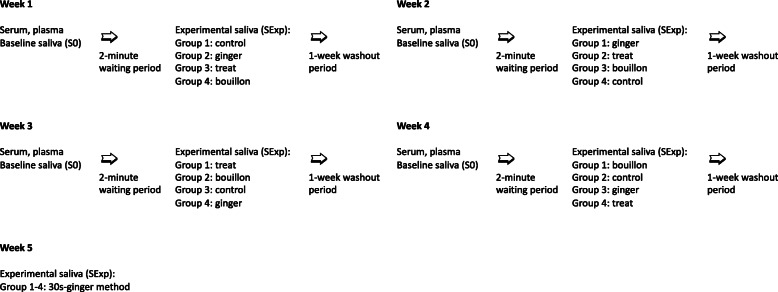
Chart illustrating the study design

A 4 × 4 randomized crossover study design was used to evaluate the differences among four salivary collection methods (Fig. 7). Each of the beagles received each of the salivary collection method with a 1-week washout period between the samplings. The order, in which the four collection methods were used, was randomly assigned. A power analysis could not be performed, as the expected differences between the salivary collection methods were not known. The investigator could not be blinded to the method used because the collector swabs looked very different.

Blood (10 ml serum (Sarstedt AG & Co, Nümbrecht, Germany) and 2 ml EDTA plasma (Greiner Bio-One, Gremsmünster, Austria)) was drawn by venipuncture from a jugular vein. Immediately thereafter, a salivary sample was collected from each dog, using a plain Salimetric® cotton swab, further defined as baseline saliva (S0). The Salimetric cotton swab was placed between the upper and lower premolars for 2 min, as previously described [10]. The dogs were allowed to chew the collecting swab to enhance saliva production and/or absorption by the cotton. After a 2-min waiting interval, a second experimental salivary sample was collected according to the group allocation, further defined as experimental saliva (SExp). One of the four collection methods was used: 1. collection with a Salimetrics® cotton swab dipped in powdered ginger (ginger group); 2. collection with a beef-flavored Salimetrics® cotton swab (bouillon group); 3. collection with a Salivette® cotton swab with a treat rolled up in it and held in the mouth with a pair of clamps (treat group); 4. collection with a plain Salimetrics® cotton swab (control group).

One week after the four experimental collection methods had been used with every dog, saliva was again collected from all 16 dogs with ginger-dipped Salimetrics® cotton swabs. This time, however, the sampling time was reduced to 30 s, instead of 2 min (SExp 30s-ginger).

#### Preparation of the cotton swabs for saliva collection

Ginger: Salimetrics® cotton swabs were dipped with 1 cm depth into a tube with commercially available ginger powder (TRS Asia’s finest foods, Ginger Powder, TRS Wholesale Co, Middlesex, England). Bouillon: beef-flavored Salimetrics® cotton swabs were prepared in advance by soaking a cotton swab in a solution prepared by mixing one beef-bouillon cube (Knorr® beef bouillon cube, Unilever Switzerland, Thayngen, Switzerland) with boiling water as per instructions on the bouillon label. The cotton swabs were then dried in an oven at 60 °C for 2 h as described by Dreschel et al. 2009 [12]. Treat: Salivette® cotton swabs were filled at the back end with a 1.5 cm piece of a commercial treat (Vitakraft® CatStick classic, Bremen, Germany) and held in the mouth with a pair of clamps.

#### Further processing of the blood and saliva samples

All blood and saliva samples were immediately placed on ice until further processing. After separation of plasma or serum by centrifugation at 1500 x g for 10 min the plasma and serum samples were stored at − 80 °C. The salivary cotton swabs were centrifuged at 1862 x g for 20 min to recover saliva. The exact amount (ul) and the pH (pH-indicator paper 1–10 Universal indicator, Merck KGaA, Darmstadt, Germany) of each saliva sample was determined before they were stored at − 80 °C. All hormone measurements of the experimental study were carried out in one batch after randomization of the samples.

#### Assessment of handling, excitement and chewing movements

Handling, excitement and chewing movements were scored always by the same investigator on a 5-point evaluation scale, with 1-very easy to 5-very difficult, 1-very low to 5-extremely high and 1-barely to 5-all the time, respectively, according to Dreschel and Grager 2009 [12].

#### Hormone measurements

Cortisol in saliva and serum was measured by use of liquid chromatography-tandem mass spectrometry (LC-MS/MS) in an ISO 17025 accredited laboratory. The minimum required sample volume for both serum and saliva was 100 μL. Saliva samples were centrifuged at 5500 X g for 15 min. The supernatant was then diluted with 500 μL water and isotopically labelled D4-cortisol (Sigma-Aldrich, Switzerland) added to a final concentration of 1.8 ng/mL and vortexed. Serum samples were also diluted with 500 μL water and isotopically labelled D4-cortisol added to a final concentration of 1.8 ng/mL. Afterwards, 250 μL of zinc sulphate (0.1 mol/L; Sigma-Aldrich, Switzerland) and 500 μL of cold methanol (− 20 °C; Sigma-Aldrich, Switzerland) were added for protein precipitation and steroid extraction, samples were vortexed and centrifuged for 5 min at 8000 g. Both serum and saliva samples were then purified using solid phase extraction on an OasisPrime HLB 96-Well Plate (Waters, UK). After loading, samples were washed 250 μL water and 250 μL of 33% methanol in water. Samples were eluted using pure acetonitrile (Sigma-Aldrich, Switzerland) and was subsequently dried under nitrogen. Samples were resuspended in 100 μL of 33% methanol in water. LC-MS/MS measurements (20 μL injection volume) were carried out by coupling of a Vanquish UHPLC to a QExactive Orbitrap Plus (both Thermo Fisher Scientific, Switzerland). Separation was achieved using an Acquity UPLC HSS T3 Column, 100 Å, 1.8 μm, 1 mm × 100 mm (Waters, UK). Mobile phases A and B consisted of water + 0.1% formic acid and methanol + 0.1% formic acid, respectively (all UHPLC grade; Sigma-Aldrich, Switzerland). Analytes were eluted using a linear gradient from 46 to 73% B over 8 min. The mass spectrometer was operated in positive ion mode using an electrospray ionization source at a resolution of 70′000.

Calibration was performed using an 8-point calibration curve ranging from 0.01 to 300 ng/mL prepared in synthetic saliva based on the recipe by Shellis [34, 35]. The analytical method for measurement of serum cortisol is based on the method described by Peitzsch at al [36]. whereas the method for salivary cortisol was originally developed and validated by us in horses [22]. To assess linearity, sensitivity, interday and intraday accuracy and precision, calibrations curves and quality control samples at four different levels in synthetic saliva (0.3 ng/mL, 3 ng/mL, 7 ng/mL, 100 ng/mL; *n* = 6 for each level) were prepared on three different days. In order to verify that accuracy and precision are also maintained in canine saliva samples, saliva samples from four dogs were additionally investigated for accuracy and precision. Lower and upper limit of quantification was defined by the lowest/highest concentration at which accuracy and precision were within 15% RSD and RSE, respectively. Linearity was assessed by the coefficient of determination (r^2^) of the calibration curve. Reproducibility in canine samples was assessed by three independent measurements of the each sample (*n* = 4), precision by standard addition to each canine saliva samples. For standard addition, each sample was spiked with three different concentration levels (0.5 nmol/L, 1 nmol/L and 2 nmol/L). To assess precision, the results obtained using the calibration curve were compared to results of standard addition experiments. Data analysis was performed using TraceFinder 4.0 (Thermo Fisher Scientific, Switzerland).

#### Test for acclimatization

To test for acclimatization to the salivary collection procedure, we compared the amount of saliva and the cortisol concentration of S0 of each week.

### Statistical analysis

Statistical analyses were performed using commercially available software (GraphPad Prism8, Graph Pad Software, San Diego, CA, USA; SPSS Statistics Version 25.0, IBM SPSS Statistics, Armonk, New York; MedCalc Software Ltd., Ostend, Belgium). Values were tested for normality by the d’Agostino and Pearson omnibus normality test. As most of the data were not normally distributed, ranges and median values are reported. Differences between groups were evaluated by Friedman’s repeated-measures test and Dunn’s post-test. The Wilcoxon signed rank test was used to examine differences between two paired measurements. The Spearman correlation was calculated for cortisol concentrations in saliva and serum. The level of significance was set at *p* < 0.05.

## Data Availability

The datasets used and/or analysed during the current study are available from the corresponding author on reasonable request.

## Abbreviations

LNSC: Late-night salivary cortisol
LC-MS/MS: Liquid chromatography tandem mass spectrometry
S0: Baseline salivary sample
SExp: Experimental salivary sample
